# Elasticity-based determination of isovolumetric phases in the human heart

**DOI:** 10.1186/1532-429X-12-60

**Published:** 2010-10-27

**Authors:** Thomas Elgeti, Mark Beling, Bernd Hamm, Jürgen Braun, Ingolf Sack

**Affiliations:** 1 Department of Radiology, Charité - Universitätsmedizin Berlin, Campus Mitte, Charitéplatz 1, 10117 Berlin, Germany; 2 Department of Cardiology, Angiology and Pulmonology, Charité - Universitätsmedizin Berlin, Campus Mitte, Charitéplatz 1, 10117 Berlin, Germany; 3 Institute of Medical Informatics, Charité - Universitätsmedizin Berlin, Campus Benjamin Franklin, Hindenburgdamm 30, 12200 Berlin, Germany

## Abstract

**Background/Motivation:**

To directly determine isovolumetric cardiac time intervals by magnetic resonance elastography (MRE) using the magnitude of the complex signal for deducing morphological information combined with the phase of the complex signal for tension-relaxation measurements.

**Methods:**

Thirty-five healthy volunteers and 11 patients with relaxation abnormalities were subjected to transthoracic wave stimulation using vibrations of approximately 25 Hz. A *k*-space-segmented, ECG-gated gradient-recalled echo steady-state sequence with a 500-Hz bipolar motion-encoding gradient was used for acquiring a series of 360 complex images of a short-axis view of the heart at a frame rate of less than 5.2 ms. Magnitude images were employed for measuring the cross-sectional area of the left ventricle, while phase images were used for analyzing the amplitudes of the externally induced waves. The delay between the decrease in amplitude and onset of ventricular contraction was determined in all subjects and assigned to the time of isovolumetric tension. Conversely, the delay between the increase in wave amplitude and ventricular dilatation was used for measuring the time of isovolumetric elasticity relaxation.

**Results:**

Wave amplitudes decreased during systole and increased during diastole. The variation in wave amplitude occurred ahead of morphological changes. In healthy volunteers the time of isovolumetric elasticity relaxation was 75 ± 31 ms, which is significantly shorter than the time of isovolumetric tension of 136 ± 36 ms (*P *< 0.01). In patients with relaxation abnormalities (mild diastolic dysfunction, *n *= 11) isovolumetric elasticity relaxation was significantly prolonged, with 133 ± 57 ms (*P *< 0.01), whereas isovolumetric tension time was in the range of healthy controls (161 ± 45 ms; *P *= 0.053).

**Conclusion:**

The complex MRE signal conveys complementary information on cardiac morphology and elasticity, which can be combined for directly measuring isovolumetric tension and elasticity relaxation in the human heart.

## Background

Times of isovolumetric contraction and relaxation and the resulting indices (e.g., Tei index) are frequently used to describe overall cardiac function [1]. The standard method for measuring cardiac time intervals is echocardiography, which exploits the opening and closing times of aortic and mitral valves [2]. Isovolumetric cardiac phases are characterized by changes in myocardial elasticity, while ventricular volume remains constant. Thus, the most direct determination of isovolumetric times combines measurement of both ventricular volume and myocardial elasticity. Cardiac volumes can be measured using ultrasound or magnetic resonance (MR) imaging. Myocardial elasticity can be measured noninvasively by elastography using either ultrasound or MR imaging and various mechanical stimuli such as time-harmonic vibrations [3-6], focused ultrasound pulses [7,8] or transient intrinsic cardiac waves [9]. In general, dynamic elastography relies on the evaluation of shear waves for recovering mechanical tissue parameters from amplitudes, wave lengths, or propagation speed. Classically, the wave equation is solved for elastic moduli using for example image-based algebraic Helmholtz inversion [10] or profile-based phase-gradient methods. Inversion techniques are well applicable in large organs such as the liver, brain, or larger groups of skeletal muscle. However, the complex anatomy of the heart requires consideration of finite geometries as proposed by Kolipaka et al. [11] and demonstrated by MR elastography (MRE) on a spherical rubber phantom. This approach assumes a lossless, isotropic, and linear elastic heart with a spherical thin-shell geometry that is vibrated solely by in-plane harmonic shear motion. Fewer assumptions are made in cardiac MRE described by Sack et al. [4], where no wave inversion is needed, and relative myocardial elasticity changes are deducible across the cardiac cycle. This wave-amplitude-sensitive MRE technique cannot measure absolute elasticity values; however, relative elasticity ratios are provided with an excellent time resolution of approximately 5 ms, which we will exploit in this study for deriving elasticity-based isovolumetric phases in the human heart. We will evaluate solely the relative timing between heart geometry and wave amplitudes based on one important observation made previously in cardiac MRE on healthy volunteers and animals: there is a distinct delay in the dynamics of wave amplitudes and ventricular volume such that the alteration of wave amplitudes begins 75 to 160 milliseconds earlier than changes in heart geometry [4,12,13]. Analyzing this timing we aim to develop wave amplitudes as a surrogate marker for alterations in myocardial elasticity during the cardiac cycle. We do not account for mechanical properties beyond shear elasticity such as viscosity, compressibility, and anisotropy or nonlinearity of elasticity. Therefore all these properties will be subsumed under the label of *apparent *elasticity, and the terms myocardial tension and relaxation will respectively refer to the increase and decrease in apparent elasticity.

In the following, the dynamics of ventricular geometry is estimated from the cross-sectional area of the left ventricle in a short-axis view of the heart. Wave amplitudes and cross-sectional areas are derived from the phase and the magnitudes of the complex MRE signal, respectively. Thus, all temporal information on the ventricular elasticity-volume cycle is available from the MRE signal. The pathophysiological significance of this information is demonstrated by applying the method to healthy volunteers and to patients with mild left ventricular relaxation abnormalities. Our hypothesis was that impaired ventricular relaxation can be directly deduced from a longer delay between the increase in wave amplitude and left ventricular dilatation at early diastole.

## Methods

### Subjects

This study was approved by the local ethics committee (EA 1/055/07-1-4), and written informed consent was obtained from all subjects. The group of healthy volunteers (*n *= 35, aged between 18 and 59 years, mean 35 ± 8.5; 4 women) had no history of cardiac disease. The group of patients (*n *= 11, aged 47-70 years, mean 60 ± 8.5; 2 women) had echocardiographically proven relaxation abnormalities (E/A < 0.75 and E/E' = 8-12).

### Cardiac MRE

Cardiac MRE was performed using clinical 1.5 T scanners (Siemens Magnetom Sonata and Avanto) and 16-channel phased-array chest coils. An ECG-gated gradient-recalled echo steady-state sequence (repetition time, *TR*, 5.18 ms; time to echo, *TE*, 3.29 ms; flip angle, α,25°; twofold GRAPPA acceleration; 128 × 96 matrix; typical FoV 320 × 250 mm; 7 mm slice thickness; 48 single-line *k*-space segments corresponding to 48 phase-encoding steps; 1.3 kHz bandwidth) was sensitized to motion by applying a sinusoidal motion-encoding gradient (MEG) of 2 ms duration, corresponding to an MEG frequency of 500 Hz. Three MRE experiments were performed on each subject while consecutively alternating the MEG orientation between read-out, phase encoding, and slice selection. The amplitude of the MEG was 25 mT/m in each direction. The start of image acquisition was controlled by the R-wave of the ECG. A total of 360 separate images were acquired across approximately two cardiac cycles (1.86 seconds = *TR *× 360). During this interval, a single phase-encoding step was performed for each image. Acquisition of all *k*-space data for each image required 48 × 1.86 seconds. During each of the 1.86-second intervals of data acquisition, the patient held their breath in expiration, followed by a short period of 2.5 seconds of free breathing (one inhalation and one exhalation) before the next *k*-line acquisition started.

A customer-built actuator produced low-frequency acoustic vibrations, which were applied to the patient's chest using a rigid piston [12]. A sinusoidal burst of 24.13 Hz frequency (1/8*TR*) was fed into the actuator after every eight *TRs*, resulting in 45 (360/8) continuous harmonic vibration cycles for each of the 48 phase-encoding steps. Depending on heart rate, one MRE scan for each vibration direction took approximately 3.5-4.0 min. Image slice orientation was set to a short cardiac axis in the anterior third of the left ventricle.

### MRE data evaluation

Images were reconstructed from 96 single-line *k*-space segments after 48 phase-encoding steps (128 × 96 matrix, 75% phase sampling, and GRAPPA factor of 2 with 12 extra lines for image reconstruction). The magnitude and phase of the complex MRE signal, *M*(**x**,t) and φ(**x**,t), were exploited for analyzing the change in left ventricular (LV) cross-sectional areas, *a_LV_*(*t*), and wave amplitudes, *U*(*t*), respectively. *a_LV_*(*t*) was determined by manual segmentation of anteroposterior and septal-lateral diameters from 90 magnitude images $\bar{M}$ averaged over four consecutive images *M*(**x**, *nTR*), *M*(**x**, [*n+*1]*TR*), *M*(**x**, [*n+*2]*TR*), and *M*(**x**, [*n+*3]*TR*) with *n *= 1,5,..360. Averaging was performed since manual segmentation of *a_LV _*required discrimination of morphological features of the heart based on the contrast of MRE magnitude images. As this contrast was inherently impaired due to the short *TR*, this type of image processing improved image quality. To further reduce physiological noise related to high *k*-space segmentation $\bar{M}$ was averaged across MRE experiments of varying motion component sensitivity. The resulting time resolution of $\bar{M}$ and *a_LV_*(*t*) was 4*TR *= 20.7 ms. Characteristic times of the cardiac cycle given by the duration of diastole (τ_dia_) and systole (τ_sys_) were determined from the *a_LV_*(*t*) function. Furthermore, the shortening fraction was calculated from the difference in *a_LV _*between end-systole and end-diastole, i.e., (*a_LV_*[end-diastole] - a_LV _[end-systole)])/*a_LV_*(end-diastole), corresponding to the shortening fraction derived from echocardiography [14].

**Figure 1:**
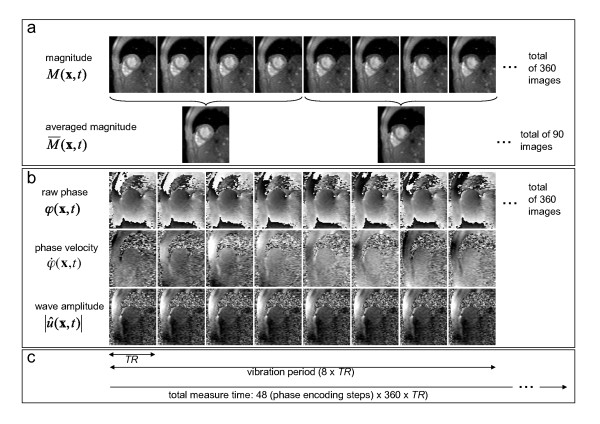
**Diagram for evaluating the complex MRE signal and deducing morphological changes as well as variations in myocardial elasticity during the cardiac cycle**. **a: **Ninety images $\bar{M}$(*t*) were obtained from 360 magnitude images *M*(*t*) by temporal averaging and used for segmenting the left ventricular cross-sectional area (*α_LV_*). **b: **Three major steps for calculating wave amplitudes *U*(*t*) from 360 raw phase images φ(**x**,*t*) as described in the text. i) unwrapping, ii) integration, iii) Hilbert transform and display as magnitude *U*(*t*). **c: **Timing of the image acquisition relative to a vibration period (see text for further details).

**Figure 2:**
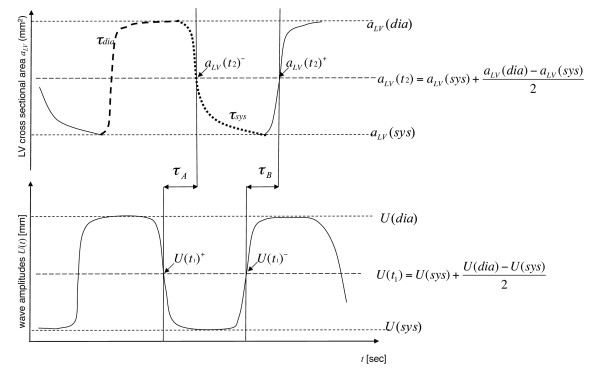
**Diagram displaying the measurements obtained from the left ventricular cross-sectional area (*α_LV_*) time curve on the top: i) duration of diastole (*τ_dia _*= dashed), ii) duration of systole (*τ_sys _*= dotted), iii) diastolic left ventricular cross-sectional area, *α_LV_*(*dia*), and iv) systolic left ventricular cross-sectional area, *α_LV_*(*sys*), and v) the resulting *α_LV_*(*t2*)^- ^on the descending systolic branch, and vi) *α_LV_*(*t2*)^+ ^on the ascending diastolic branch of the left ventricular cross-sectional area time curve**. On the bottom of the figure the measurements for the wave amplitude time curve are displayed: i) diastolic level of the wave amplitude *U*(*dia*), ii) systolic level of the wave amplitude *U*(*sys*), and the resulting iii) *U*(*t1*)^- ^on the descending branch and iv) *U*(*t1*)^+ ^on the ascending branch of the wave amplitude time curve. Isovolumetric tension time *τ_A _*and isovolumetric elasticity relaxation *τ_B _*are shown in the middle.

Calculation of wave amplitudes *U*(*t*) from the MRE phase signal φ(**x**,*t*) was performed in three major steps:

i) Calculation of the unwrapped phase velocity with φ˙=Im(exp[−iφ]∂exp[iφ]/∂t), as proposed in [4].

ii) Transformation of the resulting phase velocity to deflections with $u(\text{x},t)=q\dot{\varphi }(\text{x},t)/\omega$, where ω is the angular driving frequency. *q *(in the dimension of microns per rad) denotes the inverse encoding efficiency that scales phases to wave deflections *u *[15].

iii) Calculation of mean wave amplitudes within a region of interest (*ROI*) that is given by the outer boundaries of the left ventricle: $U(t)={\langle \sqrt{{\left|{\overset{\wedge }{u}}_{RO}(\text{x},t)\right|}^{2}+{\left|{\overset{\wedge }{u}}_{PE}(\text{x},t)\right|}^{2}+{\left|{\overset{\wedge }{u}}_{SS}(\text{x},t)\right|}^{2}}\rangle }_{ROI}$, where $\overset{\wedge }{u}$ is the complex Hilbert transform of *u*. As in other cardiac MRE studies [4,12,13], we did not exclude the blood pool from our analysis. The rationale behind this is that wall vibration is transmitted into a confined fluid by pressure or nonevanescent waves (see e.g. [16]). Figure 1 presents a diagram of how the magnitude and phase images are processed (please note that, due to averaging, the time resolution of *a_LV _*is 4 X *TR *while *U *is resolved with *TR*). Isovolumetric phases of tension, τ_*A*_, and elasticity relaxation, τ_*B*_, were deduced by means of superposed graphs of *a_LV_*(*t*) and *U*(*t*). The time shift τ_*A *_was determined by manual selection of the delay between the decaying branches of *a_LV_*(*t*) and *U*(*t*). Correspondingly, τ_B _was determined from the delay between the ascending branches of both physiological functions. More specifically, the time points *t*_1 _and *t*_2 _were selected at 50% of the maximum amplitude of the curve *U*(*t*), i.e. for *t*_1_: *U*(*t*_1_) = *U*(systole) + (*U*[diastole] - *U*[systole])/2; and for *t*_2_: *a_LV_*(*t*_2_) = *a_LV_*(systole) + (*a_LV_*[diastole] - *a_LV_*[systole])/2. Isovolumetric phases followed with τ_A _= *t*_2 _- *t*_1 _at the descending branch of both graphs (in early systole) and τ_B _= *t*_2 _- *t*_1_at the ascending branch (in early diastole). The estimation of *t*_1 _and *t*_2 _is further illustrated in Figure 2. Each time point was selected five times and averaged in order to minimize the variability of individual τ_A _and τ_B _values.

### Statistics

Data is given as mean ± standard deviation. Student's *t*-test was used to test the time intervals within each group for statistical differences. The two-sample Kolmogorov-Smirnov test was used to test the time intervals between volunteers and patients for statistical differences. Pearson's correlation test was performed to test for possible linear correlation of the measured variables. A *P*-value of < 0.05 was considered statistically significant.

## Results

In all experiments, wave amplitudes decreased due to myocardial contraction. The change in wave amplitude occurred before that in the cross-sectional area of the left ventricle.

### Volunteers

**Figure 3:**
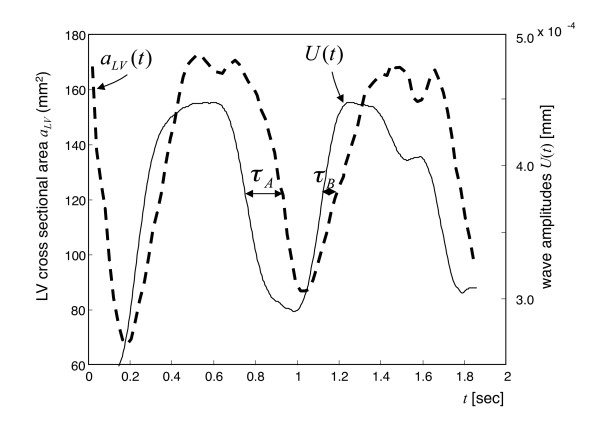
**Changes in wave amplitude *U*(*t*) and LV cross-sectional areas *α_*LV*_*(*t*) in a healthy volunteer**. Cardiac intervals of isovolumetric tension (τ_*A*_) and isovolumetric elasticity relaxation (*τ_B_*) are indicated. For further details, see text. Changes in wave amplitude *U*(*t*) and LV cross-sectional areas *α_LV_*(*t*) in a healthy volunteer. Cardiac intervals of isovolumetric tension (*τ_A_*) and isovolumetric elasticity relaxation (*τ_B_*) are indicated. For further details, see text.

Figure 3 demonstrates superposed graphs of *U*(*t*) and *a_LV_*(*t*) of a healthy volunteer and illustrates the determined τ_*A *_- and τ_*B *_-intervals. In 35 volunteers, the time of isovolumetric tension was τ_*A *_= 136 ± 36 ms. This time was found to be significantly longer than the time of isovolumetric elasticity relaxation, τ_*B *_= 75 ± 31 ms (*P *< 0.01). Similar results were obtained when accounting for normalized isovolumetric times given by τ_*A *_and τ_*B *_divided by the square root of the RR-interval [2]. Also, these normalized time intervals, τ_*A*_' = 138 ± 37 ms and τ_*B*_' = 76 ± 30 ms, were significantly different (*P *< 0.01). The shortening fraction in volunteers was 56.0% ± 5.6%. Neither τ_*A *_nor τ_*B *_showed any significant correlation with the shortening fraction, as demonstrated by low correlation coefficients, *R *= 0.34 and 0.02, respectively. The mean contractility ratio τ_*A *_/τ_*sys *_[17] of volunteers was 0.25 ± 0.06.

### Patients

**Figure 4:**
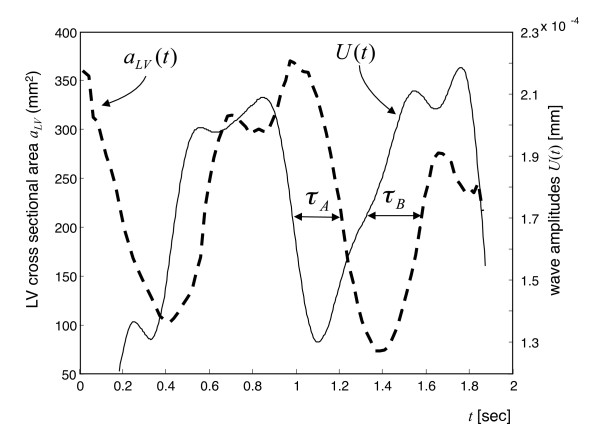
**Changes in wave amplitude *U*(*t*) and LV cross-sectional areas *α_LV_*(*t*) in a patient with LV relaxation abnormalities**. The isovolumetric tension time (τ_A_) is in the range of values measured in healthy volunteers. In contrast, the time of isovolumetric elasticity relaxation (τ_B_) was significantly increased in patients. Further explanations are given in the caption to Figure 4. Changes in wave amplitude *U*(*t*) and LV cross-sectional areas *α_LV_*(*t*) in a patient with LV relaxation abnormalities. The isovolumetric tension time (τ_A_) is in the range of values measured in healthy volunteers. In contrast, the time of isovolumetric elasticity relaxation (τ_B_) was significantly increased in patients. Further explanations are given in the caption to Figure 4.

Figure 4 shows *U*(*t*) and *a_LV_*(*t*) of a patient with mild diastolic dysfunction. The mean isovolumetric tension time in patients (τ_A _= 161 ± 45 ms) was similar to τ_A _measured in volunteers (*P *= 0.039). In contrast, isovolumetric elasticity relaxation, τ_B _= 133 ± 57 ms, was significantly prolonged in patients (*P *< 0.001). Normalized time intervals were τ_A_' = 142 ± 68 ms and τ_B _' = 173 ± 50 ms. The shortening fraction in the patient group was 44.9 ± 12.6%. Similar to volunteers, time intervals τ_A _and τ_B _showed no correlation with the shortening fraction (*R *= 0.07 and *R *= 0.29, respectively). The mean contractility ratio of patients (τ_A _/τ_sys _= 0.36 ± 0.13) was higher than in volunteers.

**Figure 5:**
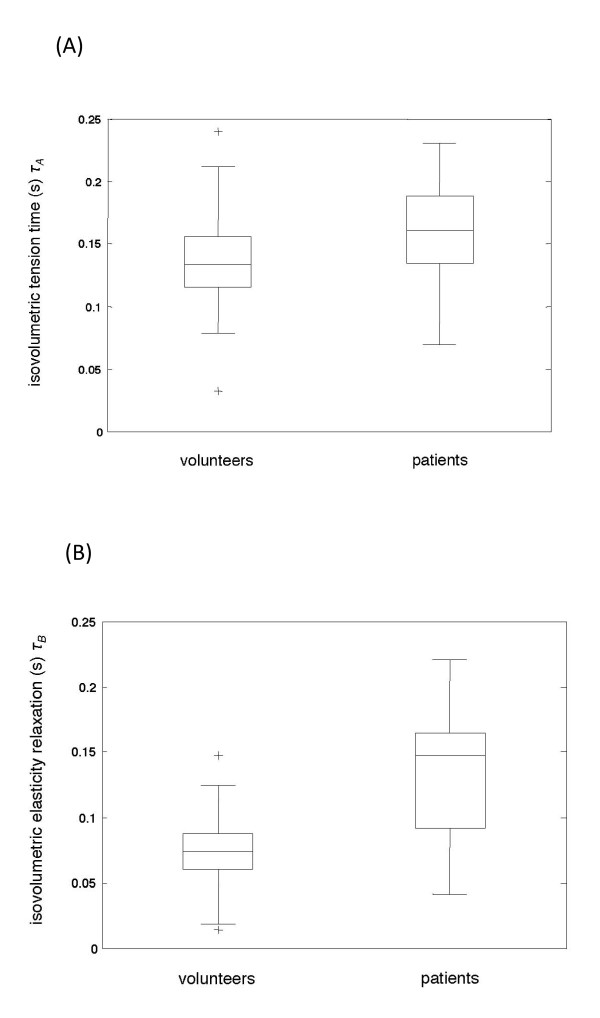
**a: Boxplot of the isovolumetric tension times (*τ_A_*) in volunteers and in patients with mild diastolic dysfunction**. The mean, the lower and upper quartiles, as well as the 50^th ^percentile (median) are displayed. The full data range is represented by the whiskers and two outliers in the volunteer group. No significant difference between the two groups can be found (mean *τ_A _*in volunteers = 136 ± 36 versus 161 ± 46 ms in patients). **a: **Boxplot of the isovolumetric tension times (*τ_A_*) in volunteers and in patients with mild diastolic dysfunction. The mean, the lower and upper quartiles, as well as the 50^th ^percentile (median) are displayed. The full data range is represented by the whiskers and two outliers in the volunteer group. No significant difference between the two groups can be found (mean *τ_A _*in volunteers = 136 ± 36 versus 161 ± 46 ms in patients). **b: **Boxplot of the isovolumetric elasticity relaxation (*τ_B_*) in volunteers and in patients with mild diastolic dysfunction. The mean, the lower and upper quartiles, as well as the 50^th ^percentile (median) are displayed. The full data range is represented by the whiskers and two outliers in the volunteer group. There are significant differences between mean *τ_B _*in volunteers (75 ± 31 ms) and that in patients (133 ± 58 ms).

All data are summarized in Table 1. Figure 5 illustrates τ_A _and τ_B _as functions of the two groups studied.

**Table 1:** MRE-derived parameters

Parameter	Volunteers	Patients	
*τ_A _*(ms)	136 ± 36	161 ± 46	*P *= 0.053, *k *= 0.44

*τ_A_*′ (ms)	138 ± 37	173 ± 50	*P *= 0.06, *k *= 0.42

*τ_sys _*(ms)	297 ± 64	365 ± 87	*P *< 0.001, *k *= 1

*τ_A _/τ_sys_*	0.25 ± 0.06	0.36 ± 0.13	*P *= 0.01, *k *= 0.54

*τ_B _*(ms)	75 ± 31	133 ± 58	*P *= 0.005, *k *= 0.55

*τ_B_*′ (ms)	76 ± 30	142 ± 68	*P *= 0.006, *k *= 0.52

*τ_dia _*(ms)	682 ± 108	517 ± 91	*P *= 0.0035, *k *= 0.61

RR interval (ms)	982 ± 102	888 ± 139	*P *= 0.08, *k *= 0.40

SF (%)	56.0 ± 5.5	44.8 ± 12.6	*P *= 0.008, *k *= 0.53

Isovolumetric tension time, *τ_A_*, and isovolumetric elasticity relaxation, *τ_B_*, measured in the left ventricle of volunteers and patients. *τ_A_*′ and *τ_B_*′ correspond to *τ_A _*and *τ_B_*, normalized by the square root of the RR-interval to account for different heart rates. *τ_sys _*and *τ_dia _*are the intervals of systole and diastole, respectively. SF denotes the shortening fraction. *P*-values relate to the difference between volunteers and patients; the Kolmogorov-Smirnov test statistic *k *is the maximum difference between the groups.

## Discussion

Cardiac time intervals give insight into the hemodynamics and mechanical function of the heart and are thus valuable indicators of cardiac health. This study for the first time analyzed cardiac intervals based on changes in elastic wave amplitudes in volunteers and in patients with mild diastolic dysfunction. The evaluation of the complex signal in MRE enabled us to derive the timing of elasticity variation in relation to that of changes in LV geometry from a single MRE examination. This paves the way for the development of software capable of automatically combining magnitude-based and phase-based information from cardiac MRE. On-site implementation would provide a new modality of cardiac MRI for the instantaneous assessment of isovolumetric times based on changes in myocardial elasticity. In this study, externally induced elastic waves were used for probing the inherent cohesion of the mechanical matrix of myocardium, which determines its macroscopic shear elasticity. Changes in shear elasticity of biological tissue are known to be sensitive to disease progression [18-20]. In particular, cardiac function is synonymous with variation in myocardial elasticity (i.e. shear modulus). A variety of heart diseases are associated with dysfunctional elasticity variations of the myocardium. An important cardiac disease pattern is diastolic dysfunction, which is characterized by an elevated elastic modulus of the ventricular wall muscle at relaxation [21-23]. This has driven the development of imaging modalities sensitive to shear modulus variations in the heart [3-9].

It is important to mention that the cardiac time intervals, τ_*A *_and τ_*B *_, analyzed in this study are not directly comparable to isovolumetric times measured by echocardiography or other more traditional methods relying on auscultation, ECG, phonocardiogram, or carotid pulse tracing [24]. Morphology-based methods allow us to assess the dynamics of heart geometry such as valve position, ventricular volumes, tissue strain, and blood flow or tissue velocity. In M-mode echocardiography the isovolumetric time intervals have been calculated from the opening and closure times of aortic and mitral valve relative to the R-wave of the ECG [25]. However, the well-known electromechanical delay limits the reliability of the time intervals deduced in this way. In Doppler echocardiography time intervals can be determined more reliably using the velocity of tissue motion [2,26]. Since both methods evaluate the change in cardiac geometry, no elasticity-based information is obtained. Hence, the time constants determined with these methods are different from τ_*A *_and τ_*B *_proposed in this study.

Isovolumetric relaxation time (IVR) measured by tissue doppler imaging is 70 ± 22 ms (healthy volunteers 30-40 years of age) [26], showing good agreement with the time of elasticity relaxation, τ_*B *_, measured in our study (75 ± 31 ms). It is known that this time interval is prolonged in mild diastolic dysfunction [27]. Surprisingly, the MRE-derived time of tension, τ_*A *_, in both groups investigated in our study (136 ± 36 ms and 161 ± 46 ms) is considerable longer than the echocardiographic isovolumetric contraction time of 63 ± 14 ms [26]. This might be attributable to the fact that the elastic properties of muscle tissue are nonlinear under large deformation, rendering the measured MRE wave response stretch-dependent [28]. Thus, the nonlinearity of myocardium results in an increase in elasticity during ventricular expansion even though the muscle is intrinsically relaxed. Upon electrical activation, the hyperelastic tension of stretched myocardium is superposed with active contraction, while the stretch-related hyperelasticity of the muscle decreases. *U*(*t*) might therefore decrease prior to systolic contraction, which would cause a longer τ_*A *_time than observed by echocardiography. Since our τ_*A *_and τ_*B *_parameters reflect the full stress-strain hysteresis of myocardium, they represent a novel measure of cardiac dynamics, whose clinical significance remains to be investigated in a large group of patients. In this study, the diagnostic significance of elasticity-based cardiac time intervals is suggested by the findings in 11 patients suffering from mild diastolic dysfunction. The fact that in this small group of patients, elasticity relaxation of the left ventricle was significantly slowed down (indicated by increases in τ_*B *_and τ_*A *_/τ_*sys*_) provides further motivation for improving the applicability and accuracy of cardiac MRE and for determining its diagnostic relevance.

## Limitations

It is known that isovolumetric relaxation time is, to a certain amount, age dependent, increasing from 74 ± 7 ms between 41 and 60 years to 87 ± 7 ms after age 60 [29], whereas the isovolumetric contraction time remains widely unchanged [26]. Therefore, further investigations are necessary to study the influence of age on our results. It is also known that, with severe diastolic dysfunction, isovolumetric relaxation time shortens, so that this parameter cannot diagnose severe diastolic dysfunction in the absence of further information [27].

Multiple factors can influence the wave amplitudes measured in cardiac MRE. We cannot discriminate shear elasticity from viscosity effects, from the reorientation of myocardial fibers that is associated with a change in the apparent elasticity (see discussion in [4]) or nonlinear effects as discussed above. A reciprocal correlation of *U*(*t*) with ventricular pressure was shown in [13]. Any further interpretation of amplitude dynamics in terms of myocardial shear elasticity is still model-based and needs experimental validation.

## Conclusion

Cardiac MRE provides temporally resolved information on both morphology and elasticity dynamics of the heart, which can be combined for determining isovolumetric times. The resulting elasticity-based timing parameters comprise tissue constitution and mechanical functioning of the heart and are thus relevant for the assessment of cardiac health. Future cardiac MRE experiments should include the evaluation of phase and magnitude information in order to further evaluate the extent to which time intervals in MRE can provide useful diagnostic information.

## Competing interests

The authors declare that they have no competing interests.

## Authors' contributions

TE, IS and BH designed the study. TE, MB and IS carried out the experiments. IS, TE and JB evaluated data and performed the statistical analysis. IS, TE and MB wrote the manuscript. TE and MB contributed equally to this manuscript. All authors read and approved the final manuscript.
